# Safety and efficacy of the automated liquid-phase embryo storage, Hatchferti: a pilot study

**DOI:** 10.7717/peerj.21703

**Published:** 2026-09-14

**Authors:** Yaming Meng, Huyu Zhou, Yanling Bai, Kangrong Gao, Junxian Jiang, Xueliang Zhou, Ming Zeng, Yuling Mao, Chunyan Wang, Jianqiao Liu, Yan Chen, Lei Li

**Affiliations:** 1 Key Laboratory for Reproductive Medicine of Guangdong Province, Guangzhou Medical University, Guangzhou, China; 2 Department of Obstetrics and Gynecology, Center for Reproductive Medicine, Guangdong Provincial Key Laboratory of Major Obstetric Diseases, Guangdong Provincial Clinical Research Center for Obstetrics and Gynecology, Guangdong-Hong Kong-Macao Greater Bay Area Higher Education Joint Laboratory of Maternal-Fetal Medicine; The Third Affiliated Hospital, Guangzhou Medical University, Guangzhou, China; 3 Guangzhou Pinjie Biological Tech Co., Ltd, Guangzhou, China; 4 Guangzhou PINZHI Medical Device Co., Ltd, Guangzhou, China

**Keywords:** Embryo storage system, Fully automation, Cryostorage, Assisted reproductive technology

## Abstract

To date, no fully automated liquid-phase embryo tank has been implemented in assisted reproductive technology (ART) laboratory and its impact on the safety and efficacy of embryo storage remains unknown. To evaluate its performance, the Hatchferti group (HG) stored a total of 230 clinically discarded 3PN zygotes (*n* = 143) and day 3 cleavage embryos (*n* = 87) from September 2023 to April 2024. These embryos were vitrified and stored for 30, 90, and 180 days, their survival and culture results were statistically analyzed. The total 3PN zygote cleavage rate after thawing was compared with the fresh 2PN zygote cleavage rate of 2023 laboratory annual data (*n* = 32,520). A 7-day period of workload data showed that the operating time in conventional liquid nitrogen tank group (CG) required for storage and retrieval were 6.71 ± 1.28 and 39.00 ± 13.42 minutes, respectively, while that in HG were significantly lower (1.95 ± 0.04 and 0.96 ± 0.03 minutes respectively, *P* < 0.05). In order to assess the stability of the operating temperature, during the 6-month study period, the temperature readings in the retrieval area were −177.84 ± 1.53 °C, and the storage area temperatures were −195.47 ± 2.01 °C and −195.81 ± 0.94 °C meeting the expected temperature for embryo storage. In the comparison between different freezing time groups, there was no statistically significant difference in the recovery rates of 3PN embryos and day 3 cleavage embryos between groups (both 100%, *P* > 0.05). There was no statistically significant difference in the cleavage rate of 3PN embryos after thawing between groups (95.56%, 95.83%, 100%, *P* > 0.05, respectively). There was no significant difference in the total zygote cleavage rate after thawing between HG (for the entire experimental period) and 2023 laboratory annual data (97.2%, 97.76%, *P* > 0.05). In conclusion, Hatchferti has demonstrated its safety and efficacy, showing promising potential for application in the ART laboratory.

## Introduction

Embryo cryopreservation is now a worldwide practice. The increasing number of frozen embryos has put forward higher requirements for reproductive centers in terms of storage and management (Christianson et al., 2020). The scattered distribution of embryos across multiple liquid nitrogen tanks leads to a burdensome workload for embryologists in terms of embryo storage and retrieval. In addition, the lack of software support and the difficulty of identifying and verifying vitrified samples may increase the risk of errors, including incorrect embryo identification (Nesbit, Porter & Esfandiari, 2022). A recent study on the time and motion of embryologists’ manual cryopreservation storage showed that each retrieval from low-temperature storage tank requires an average of 3.4 data record queries to identify the carrier, and each retrieval requires an average of 1.5 pail lifts, with each lift taking an average of 11.8  ±  9.2 s, and 47.25% of the total time spent on these storage units is considered tiring (Broussard et al., 2024). This type of labor and time-intensive work brings great physical and mental pressure to embryologists. In the current digital era, automation plays an important role in improving the efficiency, repeatability, and consistency of assisted reproductive technology (ART). Automated equipment has been applied in many ART processes, such as dish preparation, egg retrieval, semen processing, automatic intracytoplasmic sperm injection (ICSI), embryo assessment, vitrification and thawing, *etc.* (Costa-Borges et al., 2023; Liao et al., 2021; Prathalingam et al., 2022; Xiao et al., 2021; Zhu, Feng & Jiang, 2023; Zhu et al., 2023). Automated sample storage systems can provide a high level of safety and protection, and improve the management, identification, and storage chain of cryopreserved gametes and embryos, which can reduce the occurrence of events such as misidentification, misplacement, or loss of reproductive materials (Canosa et al., 2023).

To date, there is still no other automated liquid-phase embryo tank available for our selection. In this study, we have employed a new type of embryo storage system, which is also the world’s first fully automated liquid-phase embryo tank, named Hatchferti. By studying its efficiency improvement, embryo recovery and cleavage condition, we evaluate the safety and efficacy of this automated system in our center’s application.

## Materials and Methods

### Machine design and components

**Figure 1:**
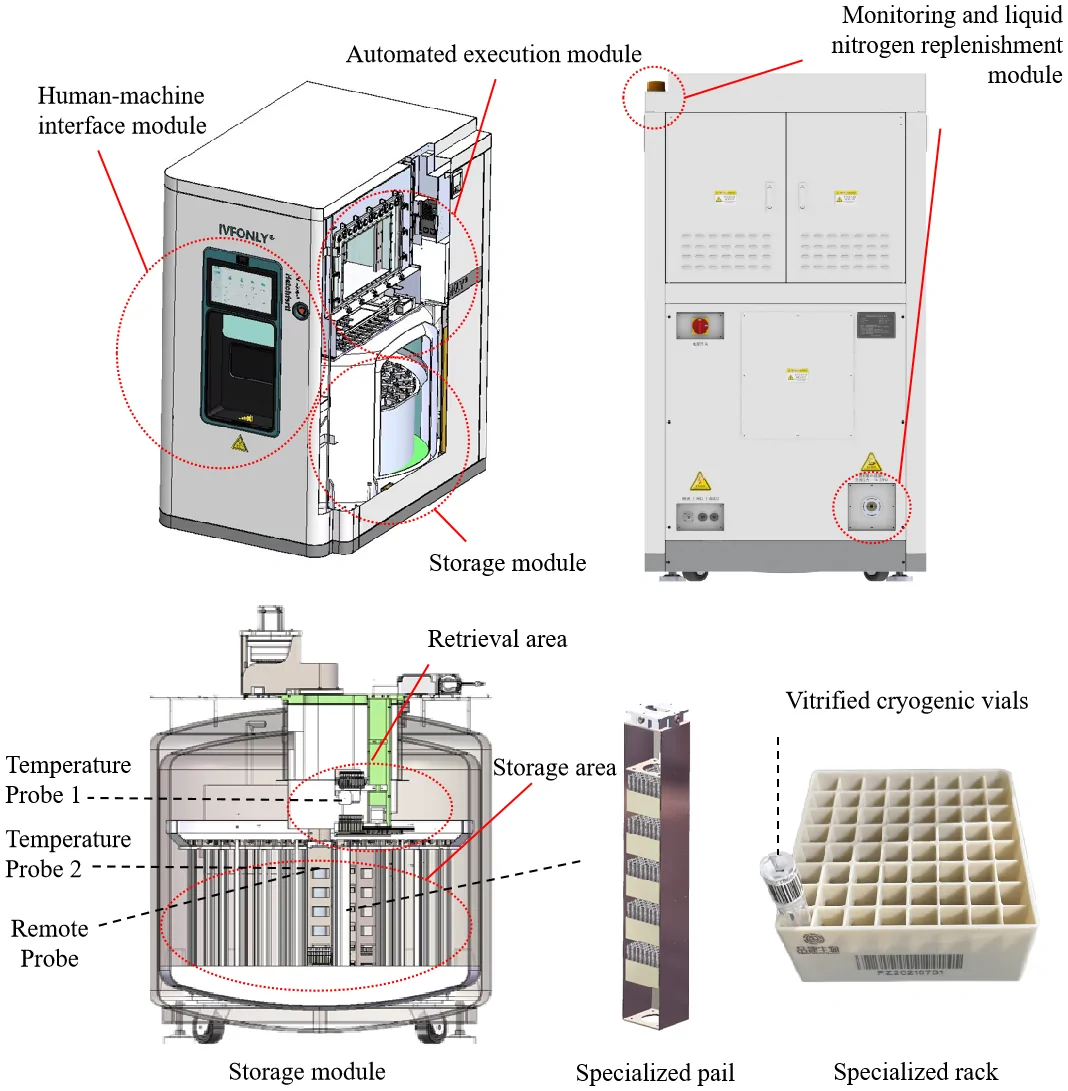
Schematic diagram of the Hatchferti.

Hatchferti is composed of four major components (Fig. 1): the human-machine interface module, the automated execution module, the storage module, and the liquid nitrogen level monitoring and replenishment module. The machine is equipped with three temperature probes inside, which are used to monitor the real-time temperature within the system. Two are located at a liquid level of 440 mm (Temperature Probe 2 and Remote Probe) and one is located in the retrieval area (Temperature Probe 1). The liquid nitrogen level is determined through data collected by the differential pressure liquid and gas phase ports and calculated by the programmable logic controller. When the liquid level falls below this minimum threshold, the replenishment module activates to automatically replenish liquid nitrogen from the Dewar flask. The device is designed to be compatible with a new type of cryogenic vials and cannot be used with traditional straws.

### Embryo storage/retrieval process

#### Fully automatic embryo system Hatchferti

**Figure 2:**
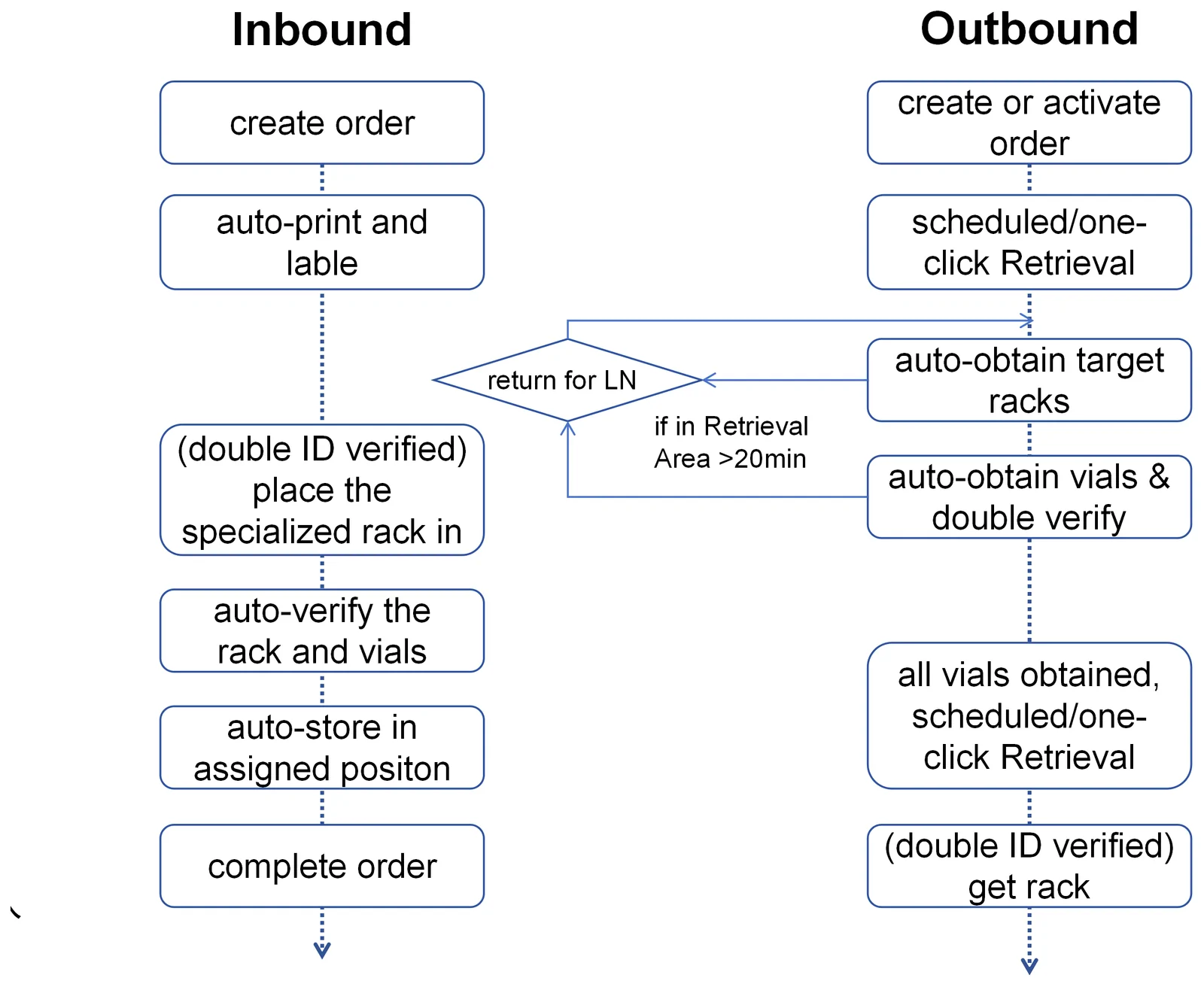
Standard inbound and outbound process for the Hatchferti.

The process of storing embryos is shown in Fig. 2. Before vitrification operations, embryologists create the corresponding storage tasks in the medical record system, print and attach labels onto the vitrified cryogenic vials (with time recorded), and then proceed with the vitrification operation. After vitrification, log in to the Hatchferti interactive screen by facial recognition, activate the corresponding task, and place the liquid nitrogen transfer bucket (containing the specialized rack and frozen sample vials to be stored) into the docking port. Inside the machine, the automated execution module completes the verification of the rack and the vials, after which the embryologist can leave the storage area, and the machine will store them in the liquid nitrogen storage area according to the specified location/storage logic. The operating time for storage process was calculated from logging in to leaving.

The process of retrieving embryos is shown in Fig. 2. Embryologists select target embryos in the system and choose schedule/immediate retrieval. Inside the machine, the automated execution module starts the retrieval. During the retrieval process, in addition to the torque sensing of the retrieval mechanism, each vial will be photographed before and after picking to verify whether it has been successfully picked (if the rack stays in the retrieval area for more than 20 min, the system will execute the return to the storage area for liquid nitrogen to ensure that the embryos are always kept in liquid nitrogen). According to the choice of scheduling/immediate retrieval, the rack will wait in the cache area or be released immediately. Embryologists can retrieve the embryos by dual facial recognition verification on the Hatchferti interactive screen. The operating time for retrieval process was calculated from logging in to leaving. Considering that our center has an annual cycle of around 7,000, the actual daily workload is relatively large. Therefore, we are considering first having experienced embryologists on rotation perform the related operations and records for a duration of 7 days.

#### Traditional manual operations

In our center, we employ an advanced vitrified cryogenic vials (details are shown in Supplemental File S1) with an Embryo Positioning Management Software (Pinzhi, China) (Li et al., 2017). The embryologist inputs patient data, documents the auto-assigned embryo positions on paper, and attaches printed labels (with printing and attaching time recorded) before cryopreservation. The vials are then meticulously placed in specific liquid nitrogen tanks and racks within the storage room by two embryologists based on the recorded information. The retrieval process is analogous, involving the identification and location of different liquid nitrogen tanks, pails, and vials based on exported information. The operating time for storage/retrieval process was calculated from entering the embryo storage room to leaving.

### Vitrification/thawing

Vitrification/thawing: The Hatchferti group consisted of clinical discarded 3PN zygotes (*n* = 143) and Day 3 cleavage embryos (*n* = 87) from our center from September 2023 to April 2024. These embryos were vitrified and stored for 30 days, 90 days, and 180 days, respectively, and their recovery and culture results (Recovery Rate = Number of Survived Embryos after Thawing/Number of Embryos Thawed * 100% (Zhang et al., 2023)) were statistically analyzed after thawing; the traditional liquid nitrogen tank group reviewed the 2023 laboratory annual data (*n* = 32,520). In the comparison between the Hatchferti group and the traditional liquid nitrogen tank group, the cleavage rate after recovery of 3PN zygote was compared to the fresh 2PN zygote cleavage rate (2PN cleavage Rate = Number of cleaved embryos Day2/Number of 2PN/2PB oocytes on Day1. ESHRE Special Interest Group of Embryology and Alpha Scientists in Reproductive Medicine (2017), 3PN cleavage rate after recovery = Number of cleaved 3PN embryos after recovery/Number of recovery 3PN). All embryos were vitrified, thawed, and cultured in the embryology laboratory according to our center’s standard protocols (Supplemental File S2). The reagents used for the procedures were products from Vitrolife (Sweden) and Kitazato (Japan), and after vitrification, they were stored in a regular liquid nitrogen tank (MVE XC47/11, USA) and Hatchferti (Pinzhi, China), respectively. The regular liquid nitrogen tank group was designated as CG, and the Hatchferti group was designated as HG.

### Statistical analysis

Data were analyzed using SPSS 26.0. Time was an indicator for evaluating efficiency.The embryo storage and retrieval time was expressed as mean  ± standard deviation and compared using the Mann–Whitney *U* test. Embryo data was an indicator for evaluating safety.Comparisons of rates between different time groups were analyzed using the Fisher exact test. Comparisons between the total 3PN zygote cleavage rate after thawing and the fresh 2PN zygote cleavage rate of 2023 laboratory annual data were analyzed using the *Z* test. *P* <  0.05 indicates statistical significance.

### Ethics

Informed consent was obtained from all individual participants included in the study. All patients provided their consent by signing a written informed consent form prior to participation. All procedures included in the study were approved by the the Ethics Committee of The Third Affiliated Hospital of Guangzhou Medical University (Ethical Review [2025] No.027).

## Results

### Manual operation time of different group

We have compiled workload data for a 7-day period (June, 2024) in our center, and the results of the time required for manual operation during the process are shown in the Table 1. During the storage and retrieval processes, the manual operation time in HG was significantly lower than that of the CG (*P* <  0.05).

### Temperature stability inside Hatchferti

During the 6-month study period, the temperature readings in the retrieval area were −177.84 ± 1.53 °C, and the storage area temperatures were −195.47 ± 2.01 °C and −195.81 ± 0.94 °C (Temperature Probe 2 and Remote Probe, Supplemental File S2, Fig. 1). Additionally, a Pearson correlation analysis was conducted on the liquid nitrogen level height and the temperature in the retrieval area, and the results are shown in Supplemental File S2, Table 1. The liquid level height and the temperature in the retrieval area showed a highly significant negative correlation (correlation coefficient of −0.912, *P* < 0.01).

### Embryo recovery and cleavage after thawing

As shown in Table 2, in the HG, the recovery rate of 3PN zygotes and Day 3 cleavage embryos was 100% for all storage durations (30, 90, and 180 days), with a cleavage rate after thawing of 95.56%, 95.83%, 100% respectively. Throughout the entire experiment, the cleavage rate after thawing was 97.20% and the cleavage rate for normally fertilized oocytes was 97.76%. There was no significant difference in the zygote cleavage rates after recovery between HG and the 2023 laboratory annual data for the entire experimental period (*P* > 0.05). As shown in Supplemental Files S2, S3, Fig. 2, there were no adverse effect on 3PN zygote and day 3 cleavage embryos rates, 3PN zygote cleavage rate after thawing in the HG.

**Table 1:** Workload data and manual operation time of different group.

	Vials to be operated^#^	Operating time in CG (min)	Operating time in HG (min)	*P* value	95%CI
Storage	32.57 ± 10.05	6.71 ± 1.28	1.95 ± 0.04^*^	0.0011	[2.98, 6.08]
Printing & Labeling	32.57 ± 10.05	3.19 ± 1.58	4.05 ± 0.15	0.3067	[−4.51, 3.57]
Retrieval	23.71 ± 9.74	39.00 ± 13.42	0.96 ± 0.03^*^	0.0011	[45.98, 51.02]

**Notes.**

* indicates a significant difference compared to the CG group.

# represents the average number of operated vials.

CG The regular liquid nitrogen tank group HG Hatchferti group

**Table 2:** The recovery rate and cleavage rate between HG and the 2023 laboratory annual data.

HG					2023 laboratory annual data	
	30d	90d	180d	Total		
3PN zygote recovery rate	100% (45/45)	100% (48/48)	100% (50/50)	100% (143/143)	/	
3PN zygote cleavage rate after thawing	95.56% (43/45)	95.83% (46/48)	100% (50/50)	97.2% (139/143)	97.76% (31,793/32,520)	Fresh 2PN zygote cleavage rate
Day 3 cleaved-embryo recovery rate	100% (27/27)	100% (30/30)	100% (30/30)	100% (87/87)	/	

## Discussion

### The effectiveness of the Hatchferti

To achieve standardization of operations and reduce variability in the *in vitro* fertilization laboratory, precise laboratory procedures and accurate training for embryologists are crucial (Casciani et al., 2021). During the vitrification thawing process, tasks such as the storage and retrieval of embryos often require highly repetitive operations. These operations are not only time-consuming but also pose potential risks to the stability of the embryo storage temperature and the safety of the operators (Tomlinson & Morroll, 2008). This study also shows that embryologists in our center need to invest about 1 h to perform the transfer tasks between different liquid nitrogen tanks and pails. In contrast, the Hatchferti system autonomously completes the complex transfer operations throughout the process, greatly freeing up the operator’s time. During the time originally used for retrieval, we can instead engage in more technical work. Moreover, the Hatchferti system allows users to pre-book the retrieval through the machine the day before, thus eliminating waiting time. On the day of thawing, simply click the retrieval button on the machine interface to quickly obtain the required samples. Similarly, during the storage process after vitrification, the operator only needs to select the storage program on the machine, place the rack in, and the machine will automatically complete the storage positioning in liquid nitrogen. In terms of the time-consuming manual operations, Hatchferti demonstrates a strong efficiency advantage. The introduction of this system not only optimizes the workflow in our laboratory but also enhances overall operational safety and reduces management difficulty.

The introduction of automated equipment not only improves operational efficiency but also significantly enhances space utilization. The commonly used cryopreservation carriers are rod-shaped carriers, which are not precise in positioning and are inconvenient to take out from the liquid nitrogen tanks. To interface with the automated Hatchferti system, a novel vial was selected for its superior space-saving properties, precise positioning, and faster handling (Li et al., 2017). The Hatchferti contains 47 specialized pails, each accommodating 5 racks that hold 64 vials each, yielding a total capacity of 15,040 samples within a footprint of approximately 2 square meters. In addition, the system optimizes sample arrangement and incorporates comprehensive inventory management functions, including automatic information verification, full-process traceability, seamless integration with electronic medical records, dual data backup with privacy protection, and hierarchical permission management, achieving virtually 100% space utilization. In contrast, a standard liquid nitrogen tank, occupying about 0.25 square meters, typically holds only approximately 800 straws and lacks automated inventory management. Vacated positions after embryo retrieval are often left unfilled, leading to low space utilization. To mitigate this, reproductive centers in China routinely consolidate vials from partially filled racks into consolidated units, freeing up racks for subsequent storage needs. However, manual inventory reorganization proves labor-intensive, time-consuming, and error-prone. Hatchferti addresses these challenges through automated vial selection coupled with real-time synchronization of embryo storage location data. By applying optimization algorithms, the system efficiently reorganizes vials between racks while maximizing available storage slots, thereby increasing storage utilization rates. Additionally, Hatchferti features an auto-replenishment function for liquid nitrogen, which eliminates the need for manual level monitoring and topping-up, thereby reducing laboratory labor and operational costs.

### Safety of the Hatchferti

The devitrification process of intracellular solutions and the surrounding extracellular medium during the thawing of cells can lead to severe damage (Macfarlane, 1986). Studies have shown that when the intracellular temperature exceeds the glass transition (Tg, which is approximately between −120 °C and −130 °C) temperature, the vitrified oocyte and embryonic cytoplasm may begin to undergo a liquid phase transition, which can promote devitrification, leading to the formation of ice nuclei and the growth of ice crystals (Jin et al., 2010). Therefore, ensuring the safety of the sample, minimizing the potential impact on the integrity of the cell structure during the cryopreservation process, and ensuring the safety and stability of the sample during long-term storage, the primary task is to maintain the temperature well below Tg. Currently, automated embryo systems using vapor-phase storage platforms have been reported (Logsdon et al., 2021). However, the literature notes that storing embryo samples in the vapor phase, although the temperature is sufficient to keep tissues below the critical glass transition temperature (around −130 °C), still requires maintaining the mechanical performance of internal devices (such as solenoid valves, heat exchangers, or fans) to safely store vitrified samples. (In an IVF laboratory in Ohio, due to a solenoid coil failure, liquid nitrogen failed to be successfully injected into the vapor tank of the container) (Pomeroy et al., 2022).

The results of this study show that in the Hatchferti retrieval area, the temperature readings are all far from the warning value (−150 °C), and all readings are below −170 °C; at the same time, the temperature in the storage area is also stable at around −195 °C. In addition, to ensure that the embryos are always stored in a liquid nitrogen environment, we have set the retention time of the vials in the retrieval area to 20 min, based on the minimum duration observed for the empty rack to deplete the liquid nitrogen in the retrieval area during previous trials (30 min, data not publicly disclosed). Through such time control, the machine can effectively ensure that the embryos are in a suitable ultra-low temperature state throughout the operation process, thereby ensuring the integrity of their structure and function. In addition, we found an interesting phenomenon that there is a significant negative correlation between the temperature in the vapor phase liquid nitrogen area (*i.e.,* the retrieval area) and the liquid nitrogen level height (Pearson = −0.912, *P* < 0.01). This finding indicates that the amount of liquid nitrogen in the tank has a significant impact on the temperature of the vapor phase area. In contrast, liquid phase storage can ensure that embryos are always stored in an environment close to −196 °C, which is far below the glass transition temperature (Tg), thus providing embryos with a more stable and safe storage condition. Moreover, studies have pointed out that there is a potential risk of devitrification when storing vitrified oocytes and embryos in the liquid nitrogen vapor phase (instead of being completely immersed in liquid nitrogen). If the operation time is too long, the embryos may enter the rubbery state and form ice crystals, leading to cell damage, thus extra caution is needed when handling samples in the vapor phase (Sansinena et al., 2014).

The results of this study show that there was no adverse effect observed after storing the discarded 3PN zygotes and day 3 cleaved-stage embryo in the Hatchferti system for 30, 90, and 180 days. These results indicate that the Hatchferti system can provide a stable storage environment for embryos, thus ensuring their safety. In this study, the safety of storing embryos and the operation procedures in the Hatchferti system was further confirmed by statistical data on the recovery rate and cleavage rate, as well as morphological analysis of embryo images. The Hatchferti system, as an efficient and stable embryo storage solution, has broad application prospects in the assisted reproductive laboratory and is expected to provide a more safe and reliable embryo storage option for infertility treatment.

The integrated software-hardware automation effectively reduces human errors, such as identification errors, location errors, or sample loss (Abdullah et al., 2023). Hatchferti manages the vial accurately through a QR code assigned and integrated with each vial. This labeling and coding system provides full traceability for the samples throughout the entire IVF process, from the initial stage to the final position in the storage tank, through a series of “matching” steps. Each key step is electronically recorded, which is not only more efficient in time compared to traditional manual recording but also significantly improves accuracy (Holmes et al., 2021). In addition, the Hatchferti employs a multi-tiered protection mechanism to ensure sample security: the temperature of the retrieval area is consistently maintained below −150 °C, with embryos perpetually immersed in liquid nitrogen within their vials. This configuration effectively isolates embryos from potential interference during concurrent handling of other vials. Regarding liquid nitrogen replenishment, the system incorporates a dual-mode control system (automatic and manual). The liquid phase in the lower reservoir maintains integrity for over 20 days until full depletion, while in the upper section remains sufficient for sample coverage for more than 4 days, providing a substantial emergency replenishment buffer. In the event of a power failure, the integrated Uninterruptible Power Supply immediately activates, sustaining system operation for ≥30 min. All samples in process during the outage are automatically returned to their designated storage positions. At the same time, the alarm information is promptly conveyed to the on-duty staff and managers through the sound and light alarm system and the software management system. Embryologists can also perform emergency handling through manual operation on the side of Hatchferti to promptly retrieve the target embryos from the system. Even under catastrophic system damage scenarios, the modular architecture based on rack design enables the complete and secure transfer of all embryos. This critical procedure can be executed without specialized equipment by a team of two operators within a 2-hour timeframe. All embryo data, operation logs, and other electronic records can also be obtained through the backup system.

While our findings are promising, the study is limited by its non-randomized design, use of discarded embryos, and a machine compatible only with cryovials. Since this experiment is a pilot trial, we are not sure whether Hatchferti will affect normal embryos. Therefore, to be on the safe side, we used the center’s annual quality control data as the control group. To address these limitations and build upon this work, a longer time prospective randomized controlled trial with normal embryos is planned, which will include long-term follow-up and a thorough assessment of economic and operational benefits. Additionally, Hatchferti is compatible with a new type of vials and is not compatible with traditional straws. Although millions vials have been used in China, widespread use globally still faces certain challenges.

## Conclusion

The automated process of Hatchferti greatly reduces the time required for manual operations and enhances the safety of operations and space utilization. In terms of safety, the Hatchferti system can effectively maintain embryos in an ultra-low temperature state. The recovery rate and cleavage rate of 3PN zygotes as well as the recovery rate of day 3 cleavage-stage embryo, stored in the Hatchferti system for 30, 90, and 180 days, are comparable to those of traditional methods, confirming the safety of storing embryos in Hatchferti. In summary, Hatchferti has demonstrated its safety and efficacy, showing promising potential for application in the ART laboratory.

##  Supplemental Information

10.7717/peerj.21703/supp-1 Supplemental Information 1 Monitoring of internal temperature of Hatchferti during the study

10.7717/peerj.21703/supp-2 Supplemental Information 2 Characteristics of the Hatchferti

10.7717/peerj.21703/supp-3 Supplemental Information 3 Vitrification and thawing protocols

10.7717/peerj.21703/supp-4 Supplemental Information 4 Statistical analysis of Table 2 Describes the detailed data, *P*-values, and 95% CIs of the statistical analysis between different groups in Table 2

10.7717/peerj.21703/supp-5 Supplemental Information 5 The pictures of 3PN zygotes and D3 cleaved-embryos in HG (200x)

10.7717/peerj.21703/supp-6 Supplemental Information 6 Raw data

## Additional Information and Declarations

### Data Availability

The following information was supplied regarding data availability: The data is available in the Supplemental Files.
