# Supplementary material for: Safety and efficacy of the automated liquid-phase embryo storage, Hatchferti: a pilot study
Source: PeerJ. 2026 Sep 14;14:e21703. doi: 10.7717/peerj.21703 (PMC13584933; doi:10.7717/peerj.21703)
Supplement: Supplementary file 1 [file peerj-14-21703-s002.docx]

**Supplemental Material 1**

**Characteristics of the Hatchferti**

Hatchferti has dimensions of 1600cm in length, 1100cm in width, and 1980cm in height. It features 47 Specialized Pails, with each pail accommodating 5 Specialized rack, leading to a total storage capacity of 15,040 cryogenic vials.

The storage units of Hatchferti are Vitrified cryogenic vials, each equipped with an internal QR code within the cap for seamless machine identification. Additionally, a label bearing patient-specific information can be conveniently attached to the circumference of the vial cap.

**Vitrified cryogenic vials (Pinzhi, China)**

Each Specialized rack can accommodate 64 vials. Throughout the procedures of depositing and retrieving the vials, the Specialized rack is filled with liquid nitrogen, which is sustained within the system for a minimum of 20 minutes, thereby guaranteeing that the embryos are continuously immersed in liquid nitrogen during all handling processes.

**Specialized rack (Pinzhi, China)**

**Vial connected with Specialized Pen (Pinzhi, China)**

**Vitrification Procedures using vials**

1. **Labeling**

Attach the label to the cap of the vial and connect it to the Specialized pen.

2. **Uploading**

After the samples are equilibrated in the vitrification reagent, load them onto the front end of the vial.

3. **Deposit**

Promptly submerge the vial in liquid nitrogen. Then fasten the cap onto the tube’s cylinder. Subsequently, apply gentle pressure to the top of Specialized pen to facilitate the placement of the tube within the Specialized rack. After all samples being vitrified, transfer the Specialized rack into Hatchferti.

**Vitrification Procedures using vials**

**Thawing procedure using vials**

Submerged the retrieved Specialized rack in liquid nitrogen, utilize Specialized pen to carefully extract the vial from it. After unscrewing the cap under liquid nitrogen environment, transfer the vial to a pre-equilibrated thawing solution to initiate the thawing process.

**Thawing procedure using vials**
