# Supplementary material for: Safety and efficacy of the automated liquid-phase embryo storage, Hatchferti: a pilot study
Source: PeerJ. 2026 Sep 14;14:e21703. doi: 10.7717/peerj.21703 (PMC13584933; doi:10.7717/peerj.21703)
Supplement: Supplementary file 2 [file peerj-14-21703-s003.docx]

**Supplemental Material 2**

**Vitrification and thawing protocols**

**3PN zygote Vitrification**

Prepare 4-6 parallel micro-drops of 20 μl each in a 100mm culture dish. Transfer the 3PN zygotes into these parallel micro-drops using a denudation pipette to sequentially wash and remove the oil. In the same dish, prepare 3 equal-sized ES micro-drops (20 μl) arranged in an equilateral triangle pattern, along with 1 separate ES micro-drop. Connect the G-Mops plus micro-drop containing the 3PN zygotes to the first ES micro-drop and let it sit for 2 minutes. Then, connect the midpoint between the two micro-drops to the second ES micro-drop and let it sit for another 2 minutes. Finally, transfer the 3PN zygotes to the last ES micro-drop and let them sit for 5 minutes. Prepare 4-6 VS parallel micro-drops in a new dish. Wash the 3PN zygotes in the micro-drops for at least 90 seconds (less than 120 seconds) before loading them, and then immerse them in liquid nitrogen.

**Embryo Vitrification**

Using a 100mm culture dish lid, prepare 3-5 ES micro-drops (30 μl) and transfer the embryos into the ES micro-drops for sequential washing. Allow the embryos to sit in the last micro-drop, with a total duration of 5 minutes. When approximately 1 minute remains in the ES solution, use another dish lid to prepare 3-5 VS micro-drops (30 μl) for sequential washing. After a minimum dwell time of 45 seconds (but not exceeding 1 minute), load the embryos and then immerse them in liquid nitrogen.

**Embryo thawing**

After removing the Vitrified cryogenic vials from liquid nitrogen, quickly place the embryo into WS1 (37°C warming stage) and let it stay for 1 minute. Then, transfer the embryos sequentially to WS2 (3 minutes), WS3 (3 minutes), and WS4 (3 minutes), performing these steps at room temperature.

Supplemental figure 1. Monitoring of internal temperature of Hatchferti during the study

Supplemental figure 2. The pictures of 3PN zygotes and D3 cleaved-embryos in HG (200x)

(a) 3PN zygote, (b) 3PN zygote after thawing, (c) cleavage stage of 3PN zygote after thawing (the second day), (d) Day 3 cleaved- embryo, (e) Day 3 cleaved - embryo after thawing

Supplemental table 1. Pearson correlation analysis of the liquid nitrogen level height and the temperature in the retrieval area

|  |  | Liquid nitrogen level | Temperature in Retrieval Area |
| --- | --- | --- | --- |
| Liquid nitrogen level | Pearson Correlation | 1 | -0.912** |
|  | Sig. (2-tailed) |  | <0.01 |
|  | n | 201 | 201 |

**indicates a significant correlation between liquid nitrogen level height and the temperature in the retrieval area.
